# Cotranslational Folding of Proteins on the Ribosome

**DOI:** 10.3390/biom10010097

**Published:** 2020-01-07

**Authors:** Marija Liutkute, Ekaterina Samatova, Marina V. Rodnina

**Affiliations:** Department of Physical Biochemistry, Max Planck Institute for Biophysical Chemistry, 37077 Göttingen, Germany; mliutku@mpibpc.mpg.de

**Keywords:** cotranslational protein folding, ribosome, polypeptide exit tunnel, nascent polypeptides, translation, protein synthesis

## Abstract

Many proteins in the cell fold cotranslationally within the restricted space of the polypeptide exit tunnel or at the surface of the ribosome. A growing body of evidence suggests that the ribosome can alter the folding trajectory in many different ways. In this review, we summarize the recent examples of how translation affects folding of single-domain, multiple-domain and oligomeric proteins. The vectorial nature of translation, the spatial constraints of the exit tunnel, and the electrostatic properties of the ribosome-nascent peptide complex define the onset of early folding events. The ribosome can facilitate protein compaction, induce the formation of intermediates that are not observed in solution, or delay the onset of folding. Examples of single-domain proteins suggest that early compaction events can define the folding pathway for some types of domain structures. Folding of multi-domain proteins proceeds in a domain-wise fashion, with each domain having its role in stabilizing or destabilizing neighboring domains. Finally, the assembly of protein complexes can also begin cotranslationally. In all these cases, the ribosome helps the nascent protein to attain a native fold and avoid the kinetic traps of misfolding.

## 1. Introduction

Proteins are a key class of biological macromolecules that are essential in all cellular processes. To execute their functions and maintain the cell viability, proteins have to fold into their specific native three-dimensional structures. Misfolding disturbs the cellular proteostasis, which can result in debilitating diseases [1,2,3]. Single amino-acid substitutions can disrupt a protein’s structure in the cell to cause, for instance, cystic fibrosis [4], sickle cell anemia [5], cataract [6], Huntington’s disease [7], or retinitis pigmentosa [8]. The molecular pathology of these diseases is a perturbation of the native three-dimensional structure leading to a misfolded protein that can no longer execute its function and is prone to aggregation and rapid degradation. Furthermore, mutations in natively disordered proteins, such as α-synuclein, tau protein or amyloid β-peptide, can cause aggregopathies, such as Parkinson’s and Alzheimer’s [2].

**Figure 1:**
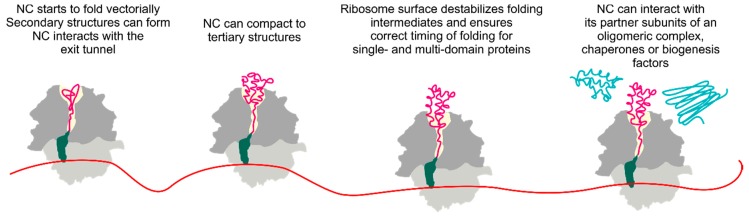
Schematic of cotranslational protein folding. Folding begins early inside the polypeptide exit tunnel. The nascent chain (NC) emerging from the ribosome can interact with chaperones, biogenesis factors, or other proteins. Small and large ribosomal subunits are shown in light and dark gray, respectively; the tRNA (green) with the nascent peptide (magenta) is shown as the ribosome moves along the mRNA (red) and the growing nascent chain moves through the polypeptide exit tunnel (light yellow). Protein partners interacting with the nascent peptide are depicted in blue.

Many proteins start to fold cotranslationally as they move through the peptide exit tunnel and emerge from the ribosome (Figure 1). About one third of the *E. coli* proteome is estimated to fold cotranslationally [9]. The average rate of protein synthesis is ~20 amino acids/s in *E. coli* [10] and ~6 amino acids/s in eukaryotic cells [11,12]. In comparison, experimentally measured rates of spontaneous folding of single-domain globular proteins range from microseconds to hours [13]. In cases where translation is slower than folding, cotranslational protein folding takes place at quasi-equilibrium conditions [14]. The ribosome can destabilize nascent folds and delay folding until the entire domain is exposed [15,16]. The vectorial nature of protein synthesis, as well as the restricted space and the physicochemical properties of the exit tunnel [17] can determine the onset of folding and define the folding landscape, thereby guiding the folding trajectory away from kinetic traps and towards stable productive conformations. The N-terminus of the emerging nascent peptide can interact with ribosome-bound chaperones, protein biogenesis factors, cofactors or partners in multi-subunit complexes, thereby ensuring correct protein localization, activity and preventing erroneous associations with proteins in the crowded cellular environment [18] (Figure 1).

Early in vitro protein refolding experiments have shown that the amino acid sequence carries all information required for small globular proteins to fold into their correct native states [19]. However, cotranslational protein folding can begin when only an N-terminal segment of the protein is available, before the C-terminal part is synthesized [20] (Figure 1). This raises the question whether the folding pathway is the same on and off the ribosome. Furthermore, large multi-domain proteins often fail to refold correctly in solution, resulting in misfolded structures and aggregation. For such proteins, domain-wise cotranslational folding may reduce the probability for off-pathway and aggregation-prone conformations [21,22], accelerate folding into the native state or even alleviate the need for chaperone assistance [20,23,24,25]. Many proteins are a part of multi-subunit complexes. These proteins not only have to adopt their individual native structures, but also to find their interaction partners in the crowded cellular environment. Cotranslational folding also plays an important role in coordinating the biogenesis of oligomeric proteins [26] (Figure 1), underscoring the importance of cotranslational events for biogenesis of different types of protein structures. A peptide emerging from the exit tunnel is monitored by ribosome-associated chaperones and protein biogenesis factors, which control folding and ensure the correct processing and cellular localization of proteins. 

In this review, we summarize current concepts of cotranslational protein folding, focusing on how the ribosome affects folding and how single-domain, multiple-domain, and oligomeric proteins fold. Other aspects of co- and post-translational folding, such as the role of chaperones and protein biogenesis factors, folding of membrane proteins, as well as the link between the rate of translation and folding, are covered by recent comprehensive reviews [27,28,29,30].

## 2. The Environment of the Peptide Exit Tunnel

**Figure 2:**
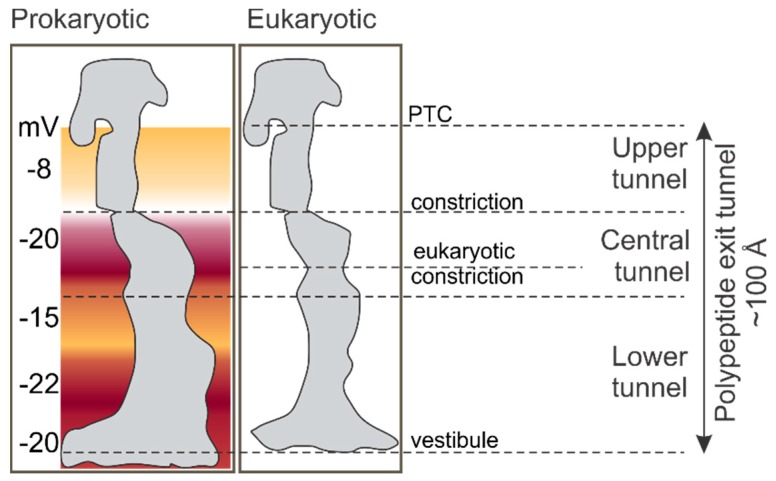
Dimensions of the peptide exit tunnel in ribosomes from prokaryotic and eukaryotic origin [34,38]. Color visualizes the electrostatic potential within the tunnel.

The peptide exit tunnel of the ribosome provides a confined space where the nascent chain begins to fold. The tunnel starts at the peptidyl transferase center (PTC) and extends for ~100 Å through the large ribosomal subunit before opening into the cytosol [31,32,33,34] (Figure 2). The tunnel is composed mainly of the ribosomal RNA (23S rRNA in bacteria and 28S rRNA in eukaryotes). Two ribosomal proteins, uL4 and uL22, of the large ribosomal subunit form a constriction of the tunnel ~30 Å away from the PTC, which is found in ribosomes from all domains of life. In addition, eukaryotic ribosomes have a second constriction formed by the extended arm of uL4 protein in the lower part of the exit tunnel [34] (Figure 2). The tunnel width varies between 10 and 20 Å and becomes wider ~50 Å away from the PTC. The last 20 Å of the tunnel form the so-called vestibule, which is generally wider than the rest of the tunnel and is shaped by proteins uL23 and uL24 in bacteria and additionally eL39 in eukaryotes (Figure 2). Residues lining the exit tunnel are highly conserved in the zone proximal to the PTC, whereas those in the vestibule have the most variation, with the tunnel in bacteria overall being wider than in eukaryotes [34]. The tunnel shields about 30–40 amino acids of the nascent peptide in the upper 80 Å of the tunnel from proteolytic digestion [35,36], although the length of the protected nascent chain may depend on the extent of cotranslational folding inside the tunnel [37].

Molecular dynamics simulations suggest that inside the exit tunnel the water is in a slowly-diffusing and semi-structured state different from the bulk or tightly bound water [39]. The water properties inside the exit tunnel may slow down diffusion and favor specific conformations of the nascent chain [39]. For hydrophobic nascent chains, the layer of water molecules between the nascent chain and the hydrophilic tunnel walls may drive nascent-chain compaction. By contrast, a polar nascent chain in this same situation would experience a smaller drive to form helical structures, but would rather displace the ordered solvent molecules on the surface of the tunnel resulting in close contact between the nascent chain and the tunnel walls [39]. Biochemical and structural studies suggest that nascent chains may form helical structures in the upper regions of the tunnel, even though a peptide with the same amino acid sequence in solution does not form a stable helix [37,40,41]. 

Aside from restricting the folding space, the peptide exit tunnel provides a characteristic electrostatic environment. Ribosomal proteins and rRNA that line the tunnel walls contribute to the global electrostatic potential of the tunnel. On average, the tunnel is more negatively charged than the cellular matrix [38]. The charge is unevenly distributed and varies from −8 mV to −22 mV along the length of the tunnel [38] (Figure 2). The lowest potential, −20 mV, is found at the constriction near the uL4 and uL22 proteins. The high degree of conservation of the rRNA sequence and of the charged amino acids lining the tunnel walls suggests that the electrostatic properties of the tunnel are functionally important [34]. In fact, experiments with ribosomal protein S6 as a model nascent chain suggest that changing the charge distribution along the nascent peptide sequence by introducing mutations affects the rate of cotranslational folding, and the more positive the net charge of the protein, the deeper in the exit tunnel it is folded [42]. The combination of the tunnel geometry and electrostatic potential imposes restrictions that define the size, the complexity, and the timing of folding intermediates. Perturbations in the shape of the tunnel caused by deleting tunnel-exposed loops of uL23 and uL24 shift the onset of cotranslational protein folding, for example of proteins ADR1, R16, and I27 [43]. 

Nascent chains can interact with the peptide exit tunnel in specific ways that affect the rate of translation. Stretches of positively charged residues can slow down [44,45] or even stall [46] translation. Changes in translation rates can affect the rate of folding and the conformation of the resulting proteins [47,48]. Some peptides, such as those found in SecM, MifM, VemP, ErmCL, cause programmed translation arrest, thereby regulating the expression of the respective downstream genes [49]. These arrest peptides (AP) are usually ~20 amino acids long; they interact with the exit tunnel and distort the optimal geometry of the PTC [49,50]. In some cases, stalling brings into the PTC a pair of slowly reacting amino acids, such as proline and glycine that do not react with one another unless the active conformation of the PTC is induced. The AP of SecM is of particular interest [51,52,53]. When fully translated, the 17 amino acid SecM AP inhibits peptidyl transfer until an external force exerted on the nascent peptide alleviates stalling, allowing the ribosome to resume translation [53]. Cotranslational folding events can exert mechanical force of up to 8 pN) [54,55] and relieve AP stalling thereby allowing translation to continue. This is utilized in force-profile assays (FPA) to identify cotranslational folding events [51,52,53]. 

## 3. Folding inside the Exit Tunnel

**Figure 3:**
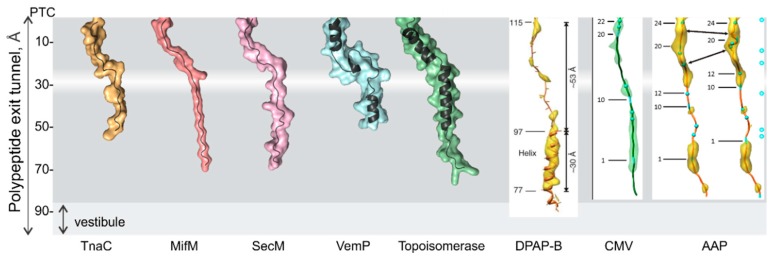
Examples of structures of nascent peptides in the polypeptide exit tunnel. Nascent peptides can interact with the tunnel walls as shown for TnaC [68], MifM [69], SecM [70], and CMV [71], or form α-helices in the upper and lower regions of the tunnel, as illustrated for VemP [62] and DNA topoisomerase peptides [61]. An α-helical structure of dipeptidylaminopeptidase B (DPAP-B) and the AAP peptide in the tunnel are also shown [60,71]. Structures shown on gray background are visualized using the PDB coordinates (PDB ID left to right: 4UY8; 3J9W; 3JBU; 5NWY; 5NP6). The coordinates of structures shown on white background are not available as PDB entries and are reproduced from the respective journals, with permission.

Early experiments using fluorescence resonance energy transfer (FRET) between labels attached at different positions in the nascent peptide suggested that transmembrane segments can form α-helices within the exit tunnel in the proximity of the PTC [40]. Biochemical assays based on site-specific cysteine tagging (pegylation) of the nascent chain helped to establish that the secondary structure formation can happen in a tunnel zone proximal to the PTC or at the distal end of the tunnel [56,57,58,59]. Visualization of nascent chains by cryo-electron microscopy (cryo-EM) shows that α-helices can form in the upper and lower regions of the tunnel [51,60,61,62], whereas the space at the constriction is too narrow to accommodate an α-helix (Figure 3). However, not every polypeptide chain that ultimately adopts helical conformation starts folding inside the peptide exit tunnel. The overall hydrophobicity, propensity to form an α-helix, and the element length are the major determinants of α-helix formation within the tunnel [63]. Indeed, accessibility assays, FRET, and molecular dynamics simulations provide evidence that transmembrane helices favor early compaction during translation to a much larger extent than their soluble counterparts [40,63,64]. 

**Figure 4:**
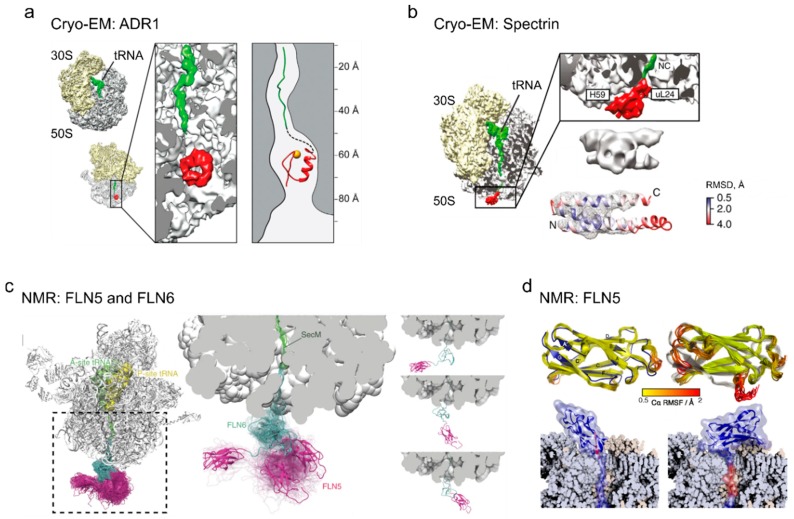
Tertiary structures of nascent peptides on the ribosome (adapted with permissions). (**a**) Cryo-electron microscopy (cryoEM) structure of the Zn-finger domain of ARD1 deep inside the exit tunnel of the ribosome. Figure adapted from [51]. (**b**) Cryo-EM structure of partially folded states of the spectrin domain at the exit tunnel vestibule, adapted from [16]. Root-mean-square deviation (RMSD) indicates the deviation of the native spectrin domain structure (PDB: 1AJ3) from the cryo-EM density map (EMD-3451) of the domain conformation at the ribosome surface. (**c**) Nuclear magnetic resonance (NMR) structures of the disordered FLN6 domain (cyan) and natively folded FLN5 (magenta), with representative conformations of FLN5 on the ribosome; figure adapted from [82]. (**d**) NMR structures of the native state (left) and an ensemble of intermediate states (right) for FLN5 on the ribosome [78].

Nascent chains can also form tertiary interactions within the exit tunnel of the ribosome [59,65] and molecular dynamics simulations predicted a number of domain structures that could fold in the tunnel vestibule [66]. FPA reveals that small protein domains with a molecular weight <10 kDa (or ≤70 amino acid residues) of various topologies encompassing α-helices or β-sheets may fold within the first 80 Å of the exit tunnel [42,67]. FPA and cryo-EM show that an entire Zn-finger domain of ADR1 folds into a native structure deep inside the exit tunnel of the ribosome [51] (Figure 4a). Also, the α-helical N-terminal domain of HemK forms a compact intermediate deep within the exit tunnel, although the native fold is attained only upon leaving the ribosome [37]. These examples also show that in some cases the tertiary interactions formed inside the tunnel can be very similar to the native structure of the isolated fully folded protein [51], whereas others are strictly cotranslational and not observed during protein refolding in solution [37]. The onset and trajectory of folding may be determined by the relative stability of the first accessible folding intermediate, rather than by the specific biophysical properties of the isolated native protein [16] (Figure 4b).

## 4. Cotranslational Folding of Single Domain Proteins

The ribosome can define a unique folding trajectory of single-domain proteins by inducing formation of simple folding units/intermediates early during translation. For example, a small globular N-terminal domain of protein HemK that is a rapid two-state folder in solution undergoes gradual compaction on the ribosome [37,72,73]. Likewise, spectrin domain, which is a two-state folder in solution, begins to fold cotranslationally before the C-terminus becomes available and proceeds via an ensemble of partially structured states [16,74] (Figure 4b). Fluorescent proteins GFP and RFP cannot fold into the native state while the C-terminus is occluded by the ribosome, but the proteins remain in a compact folding-competent non-native conformation [75]. The nucleotide binding domain of cystic fibrosis transmembrane conductance regulator (CFTR) folds through a series of precisely timed and controlled nascent chain compaction events that are different from its folding trajectory in solution, which is facilitated by the ribosome through optimized translation kinetics [76]. The binding of its ligand, ATP, to the N-terminal domain stabilizes an energetically favorable local conformation, thereby contributing to the folding trajectory [77]. 

Somewhat surprisingly, also β-stranded domains can initiate folding on the ribosome via pathways that differ from those in solution. Upon cotranslational folding of the FLN5 filamin domain, the first intermediate is formed deep inside the exit tunnel, as found by FPA [73], although nuclear magnetic resonance (NMR) experiments suggest that this compaction is not identical to the final native fold and the protein appears unfolded. The protein then undergoes a transition to the native state after emerging from the exit tunnel [78,79] (Figure 4c,d). Proteins containing repeat motifs can also fold sequentially. FPA reveals that a β-helix pentarepeat protein folds through at least four cotranslational intermediates, which are attributed to the stepwise compaction of the first several coils, followed by a compaction when the entire domain emerges from the exit tunnel [80]. In the cases where the ribosome induces early cotranslational folding, rapid initial compaction of the N-terminal elements of the nascent chain can form the nucleus for further cotranslational folding. For complex domain topologies, the establishment of a stable folding nucleus on the ribosome ensures that a protein packs into conformations that do not lead to misfolding or aggregation [81]. Destabilization of the native domain in these cases does not change the onset of folding [16,81]. The folding trajectory is defined by the stability of folded or partially folded states formed on the ribosome, whereas the stability and folding rates of isolated native proteins are insufficient to predict the cotranslational folding pathway [16]. 

There are also examples where the ribosome prevents folding until a large part of the domain emerges in the cytoplasm. Some small globular proteins that can rapidly refold from unfolded to native state in solution remain unfolded during translation and adopt their native-like assembly only when most of the peptide has emerged from the exit tunnel [83]. NMR studies of truncated SH3 peptides of various length show that on the ribosome they remain flexible and unstructured, but once the entire domain sequence emerges out of the tunnel, it folds into a compact, native-like β-sheet assembly [83]. Phi-value analysis [84], which allows one to estimate the contribution of each amino acid residue to the rate-limiting transition state on the protein folding pathway, suggests that the ribosome does not change the key contacts required for the transition towards the native structure of all β-sheet Ig domains of titin I27 [85] or SH3 domain [86]. In some cases the ribosome has no effect on folding. For example, the intrinsically disordered protein α-synuclein is not perturbed on the ribosome, despite the interactions established between nascent protein and ribosome [82]. 

Cotranslational folding intermediates may have biological roles on their own. A structurally unique cotranslational intermediate of FtsY determines its targeting to the membrane [87]. During translation, a specific FtsY domain forms an extended helix that reorganizes into the final three-helix bundle only after the fully translated nascent chain is released from the ribosome. The extended conformation does not exist in the fully folded native protein, but is thought to facilitate the cotranslational localization of FtsY [87]. 

Coarse grained molecular dynamics simulations of co-translational folding for 5 different globular proteins suggest that the cooperativity of folding decreases on the ribosome due to the appearance of partially folded N-terminal intermediates that are not populated in solution. The ribosome decreases the diversity of the folding routes and increases the probability of folding beginning from the N-terminus [88]. Monte Carlo simulations of cotranslational folding processes suggest that during elongation the ribosome may support the progressive establishment of structures that are dominated by local interactions, whereas protein structures that are governed by more distal interactions do not fold until the nascent chain is released into solution [89]. Interestingly, there is some structural evidence that shows that truncated forms of a β-sheet protein in isolation may adopt an α-helical conformation and undergo a conformational transition to the antiparallel β-sheet topology only when a sufficient length of the peptide chain is synthesized [90]. Although the latter work was carried out with protein fragments in solution, rather than with translating ribosomes, it points towards the idea that short-range interaction may be favored in early cotranslational intermediates, but as the peptide grows, the structure rearranges to establish the final long-range contacts. Interestingly, interaction with the translating ribosome may even coordinate the formation of the knot in the so-called knotted proteins [91,92]. Course grain simulations suggest that the nascent twisted loop sticks to the ribosome surface and is threated by the C-terminal part of the chain being pushed out of the ribosome, with the creation of the knot [91].

## 5. Multidomain Protein Folding

About 30–40% of proteins in prokaryotic and up to 75% in eukaryotic cells are multidomain proteins [93]. During refolding experiments in solution, multidomain proteins tend to misfold and form insoluble aggregates [94,95,96]. In the cell, the ribosome and the chaperones ensure the correct folding trajectory [97]. Systematic studies of protein coding sequences show that slowly translating codon clusters frequently occur at domain boundaries [30,48] suggesting that individual domains might be folding at least partly independently of one another starting from the N-terminal domain and proceeding in a vectorial fashion as each subsequent domain is synthesized. For example, the N-terminal domains of HemK and CFTR fold largely independently of the C-terminal part of the protein [37,98]. Similarly, in mammalian cells folding of multidomain fusion protein rapamycin binding protein (FRB)-GFP occurs co-translationally and strongly favors a domain-wise folding pathway [99]. 

There are only a few examples of multidomain proteins for which the cotranslational folding pathway is known. The only rigorously studied case is EF-G, a five domain translation factor that binds GTP. In isolation, EF-G refolds very inefficiently, both on the level of individual domains and of a complete protein, suggesting that the domains can form non-native off-pathway intermediates that preclude refolding to the native structure [100]. In contrast, in vivo EF-G folds all five of its domains into a functional conformation. On the ribosome, the N-terminal G domain of EF-G folds autonomously, but the nascent domain structure remains unstable [100], delaying folding until sufficient sequence information is available, or the subsequent domain/interaction partner becomes available for interaction. The folding of the G domain must occur before the folding of the next domain (domain II of EF-G). The ribosome can either accelerate or decelerate the folding of the G domain, compared to the rate of folding in solution, depending on how much amino acid sequence has been synthesized. The maximum folding rate of the nascent G domain is achieved when the nascent chain comprises 386 amino acids; at shorter peptide lengths the ribosome decelerates G domain folding, while for longer peptides the rates of folding on the ribosome are higher than in solution. The timing of the individual domain folding is crucial, because the interaction between the folded and unfolded domains in EF-G can result in unfolding of parts of the structure on the ribosome, thereby further complicating the cotranslational folding landscape [100,101]. In this case, the ribosome, together with trigger factor (TF), a cotranslationally acting chaperone, aids early folding steps to establish the correct path for folding [101]. Notably, in vivo folding of the eukaryotic homolog of EF-G, eEF2, requires the help of chaperones [102,103]. Interestingly, recent work on the cotranslational folding of domain III of EF-G shows that this domain is not stabilized by its N-terminal neighbors (domain G and domain II) and requires interactions with the C-terminal domains (domains IV and V) to adopt a stable structure [104]. This is probably related to the fact that domains G+II and III+IV+V form the two superdomains of EF-G that move relative to each other during its function in translocation. These data also imply that about halfway through synthesizing EF-G, the folding pathway shifts from cotranslational to post-translational. The high degree of flexibility in domain III is required for EF-G to execute its function, but this feature leads to an increase in the number of unfolded domains during synthesis on the ribosome. This illustrates how different biological requirements have to be reconciled during protein biogenesis [104]. 

## 6. The Ribosome Has a Destabilizing Effect on the Nascent Chain

In addition to promoting correct protein folding, the ribosome prevents premature or incorrect folding. The surface of the ribosome destabilizes the nascent protein packing even after the proteins have fully emerged from the exit tunnel (Figure 1). The examples of the ribosome acting as a holdase are during the synthesis of FLN5 [82], RnaseH [105], T4 lysozyme [15], GFP and RFP [75]. Optical-tweezer experiments on the cotranslational folding of T4 lysozyme show that the nascent protein interacting with the ribosome surface has a different rate of folding than in solution. Changing the ionic strength of the buffer affects the protein folding rate on the ribosome, suggesting that electrostatic interactions between the peptide and the negatively charged ribosome surface are responsible for this effect [15]. In other cases, the ribosome can delay the formation of cotranslational intermediates at the emerging N-terminus, disfavor the formation of misfolded intermediates and increase the rate of their unfolding in order to maintain a folding-competent nascent polypeptide [106]. Delaying the compaction of nascent chains could be advantageous in ensuring that folding into stable conformations does not occur before the entire sequence is fully accessible. The fine-tuning of the folding window could be of particular importance for cotranslational folding of multidomain proteins, where interactions between unstable folding intermediates can derail folding of the entire protein [100,101]. The highly negative electrostatic charge of the ribosome surface may help in achieving these destabilizing effects. Modulating the net charge of an intrinsically disordered protein alters the population distribution of the dynamic nascent chain species on the ribosome: the higher the net negative charge of the nascent chain, the larger the fraction of the more dynamic population of the nascent chain on the ribosome [107]. Coarse-grained molecular dynamics simulations of several globular proteins attached to the ribosome by a linker of different length suggest that at the ribosome surface the entropy of the unfolded state increases and that of the native state decreases, causing destabilization of the nascent protein structure. The unfolding rates decrease and the folding rates increase linearly with the increasing linker length, which explains why native folds are stabilized as the protein moves away from the ribosome [88].

## 7. Cotranslational Subunits Assembly

Prokaryotic genomes are organized in operons where a single mRNA encodes multiple protein products. The individual subunits of protein complexes tend to be encoded within the same operon, and the order of genes in an operon is non-random and under selective evolutionary pressure [108]. Recent studies suggest that this may be maintained in part because the assembly of multisubunit proteins can begin cotranslationally. An elegant study employing the bacterial luciferase LuxAB shows that when both subunits are synthesized from a single bicistronic mRNA, LuxA binds to the nascent LuxB before the latter is released from the ribosome [109]. The extensive heterodimer interface between the two subunits is established as soon as the entire dimerization surface of LuxB emerges from the exit tunnel. Cotranslational assembly of multisubunit complexes is one of the most effective ways to ensure rapid and efficient recruitment of partner proteins in the crowded environment of the cell. 

A significant fraction of eukaryotic proteins form large protein complexes [110]. mRNAs encoding the protein subunits of an oligomeric complex in eukaryotic cells are often colocalized [111], possibly to bring together interacting protein partners. However, even high local protein concentration cannot explain the high efficiency of protein complex assembly. Indeed some proteins are by themselves toxic to the cell [112] or unstable, intrinsically disordered and prone to aggregation [2,113]. Recent studies suggest that several mammalian nuclear transcription complexes assemble cotranslationally [114]. A systematic study of eukaryotic subunit assembly during translation by selective ribosome profiling shows that out of 12 hetero-oligomeric complexes studied, nine assembled cotranslationally and the remainder assembled with chaperone assistance [26]. In most cases, the uni-directionality of cotranslational assembly is evolutionarily preserved from prokaryotes to eukaryotes and the onset of subunit interaction coincides with the emergence of the interaction domain of the nascent peptide. Cotranslational association is favored in those cases where subunits are especially aggregation-prone [26]. Also yeast protein complexes, such as histone-modifying complexes methyl-transferase (SET1C) [115] and acetyltransferase (SAGA) assemble cotranslationally [116], as do cyclin protein complexes [117]. The ribosome may modulate the assembly of protein complexes by stabilizing individual protein domains or subunits [100] or adjusting the speed of translation [47] downstream of interaction domain boundaries [30]. This may help to find the optimal time window for interactions between the protein subunits. The electrostatic charge of the ribosomal surface can also act in regulating cotranslational subunit assembly. For example, intrinsically disordered proteins of opposite charge, ACTR and NCBD, form a complex on the ribosome cotranslationally, but only with ACTR as the nascent chain and NCBD free in solution, and not vice versa. The negatively charged nascent ACTR is repelled from the negatively charged ribosome surface and thus remains available for productive binding of its positively charged partner [118]. The repulsion of negatively-charged nascent chains is consistent with previous observation of increased dynamics of negatively charged nascent chains on the ribosome [107]. A picture emerges where cotranslational assembly of subunits depends on multiple features of proteins, ribosome and the cellular environment; it could thus be subject to regulation on multiple levels to maintain the proteostasis in the cell.

## 8. Future Perspectives

Recent work has shed new light on the mechanism and relevance of the cotranslational folding of nascent proteins. The major conclusion is that folding of many proteins is governed by the ribosome depending on the intrinsic properties of the nascent peptide, such as its type of fold, size, thermodynamic stability, surface charge, and function. Understanding the physico-chemical rules that govern cotranslational folding is one of the future challenges towards solving the folding problem. Because protein synthesis is an energetically costly process, the optimal interplay between translation kinetics and cotranslational folding can ensure efficient protein production. This makes the ribosome a key player in maintaining protein homeostasis in the cell, but also raises questions concerning the links between translation and folding. For example, folding has been suggested to affect the rate of translation, but experimental evidence for this is scarce. It is known that translation is a non-uniform process, but the reasons for ribosome pauses, the interaction between adjacent ribosomes in polysomes, and the understanding of the role of these translational events for protein folding has just started to emerge. To be able to make generalizations, we need more examples of how multidomain and oligomeric proteins fold. One puzzling question is how the ribosomes synthesizing the subunits of a multidomain complex colocalize to start the cotranslational assembly. Growing evidence suggests that the ribosome acts as a holdase for nascent proteins. However, it is unclear how the interactions of the downstream effectors, such as chaperones and protein biogenesis factors, shape the nascent protein-folding trajectory. One can expect that this will be among the major future questions for the years to come.
